# Submillimeter-Long WS_2_ Nanotubes: The Pathway to Inorganic Buckypaper

**DOI:** 10.1021/acs.nanolett.3c02783

**Published:** 2023-10-08

**Authors:** Vojtěch Kundrát, Rita Rosentsveig, Kristýna Bukvišová, Daniel Citterberg, Miroslav Kolíbal, Shachar Keren, Iddo Pinkas, Omer Yaffe, Alla Zak, Reshef Tenne

**Affiliations:** † Department of Molecular Chemistry and Materials Science, Weizmann Institute of Science, Rehovot 7610001, Israel; ‡ Thermo Fisher Scientific, Vlastimila Pecha 12, CZ-62700 Brno, Czech Republic; § Central European Institute of Technology, Brno University of Technology, Purkynova 123, CZ-61200 Brno, Czech Republic; ∥ Institute of Physical Engineering, Brno University of Technology, Technická 2, 616 69 Brno, Czech Republic; ⊥ Department of Chemical and Biological Physics, Weizmann Institute of Science, Rehovot 7600001, Israel; # Department of Chemical Research Support, Weizmann Institute of Science, Rehovot 7600001, Israel; ¶ Faculty of Science, Holon Institute of Technology, Golomb Street 52, Holon 5810201, Israel

**Keywords:** tungsten disulfide nanotubes, tungsten suboxide nanowhiskers, growth, sulfidation, buckypaper, felt, wet-laying

## Abstract

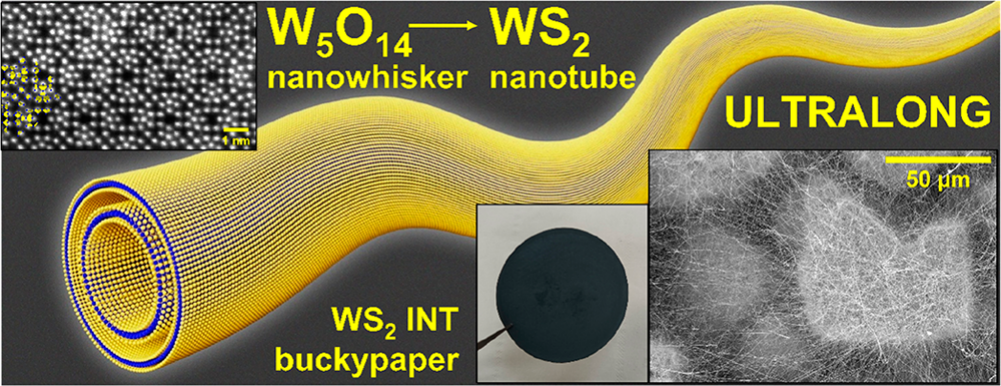

WS_2_ nanotubes present many new technologies under development, including reinforced biocompatible polymers, membranes, photovoltaic-based memories, ferroelectric devices, etc. These technologies depend on the aspect ratio (length/diameter) of the nanotubes, which was limited to 100 or so. A new synthetic technique is presented, resulting in WS_2_ nanotubes a few hundred micrometers long and diameters below 50 nm (aspect ratios of 2000–5000) in high yields. Preliminary investigation into the mechanistic aspects of the two-step synthesis reveals that W_5_O_14_ nanowhisker intermediates are formed in the first step of the reaction instead of the ubiquitous W_18_O_49_ nanowhiskers used in the previous syntheses. The electrical and photoluminescence properties of the long nanotubes were studied. WS_2_ nanotube-based paper-like material was prepared via a wet-laying process, which could not be realized with the 10 μm long WS_2_ nanotubes. Ultrafiltration of gold nanoparticles using the nanotube-paper membrane was demonstrated.

Metal dichalcogenide nanotubes, and in particular those made of WS_2_ and MoS_2_, have been known for over 30 years^1^ and have been investigated extensively both experimentally and *in-silico*. They usually come in multiwall form with a diameter of 20–150 nm and aspect ratio ∼100. The synthesis of macroscopic amounts of such nanotubes was described by a number of authors.^2−10^ A new synthetic approach for achiral multiwalled WS_2_ nanotubes using gold nanoparticles as growth promoters was recently described.^11^ Their optical and electrical properties have been investigated in some detail recently. WS_2_ nanotubes were found to exhibit a superconducting transition at 5.8 K,^12^ also exhibiting Little–Parks oscillations. A strong bulk photovoltaic effect was observed in such nanotubes, which was also attributed to the inherent loss of time reversal symmetry in such chiral nanotubes.^13^ Several other significant results in this field are the observation of strong coupling between optical cavity modes and the excitons in MoS_2_^14^ and WS_2_^15^ nanotubes, second harmonic generation,^16^ and sliding ferroelectricity, which was exploited for recording optical information.^17^ Single-level quantum transport was observed at temperatures below 100 mK for MoS_2_ nanotubes with bismuth contacts.^18^ Self-sensing torsional resonators based on WS_2_ nanotubes were also recently demonstrated.^19^

WS_2_ nanotubes were found to exhibit a major reinforcing effect on polymer nanocomposites.^20−22^ In particular, given their nontoxic behavior,^23^ such nanotubes could be utilized for reinforcement of biocompatible polymers.^24−26^ It is well documented that the reinforcement effect of such 1D nanostructures increases with their average aspect (length/diameter) ratio,^27^ making the present research particularly interesting.

The shape and morphology of the WS_2_ nanotube are driven by the dimensions of the precursor nanowhisker.^3,28^ Using a fluidized bed reactor some small fraction of WS_2_ nanotubes up to 0.5 mm long were reported.^29,30^ However, the reaction conditions were ill-controlled and the fraction of long nanotubes from the entire product was very small. Using a horizontal flow reactor, pure phase of nanotubes several micrometers long with an aspect ratio of 100 were obtained.^31^ In the present study, ultralong W_5_O_14_ nanowhiskers were obtained first in high yield via a reaction in a sealed ampule. The oxide nanowhiskers were subsequently sulfidated in a flow reactor ultimately producing almost pure phases of ultralong (0.1–0.5 mm) WS_2_ nanotubes with an aspect ratio >1000 in high yield. This new work paves the way for the controlled synthesis of ultralong WS_2_ nanotubes in appreciable amounts. Moreover, the electrical properties and photoluminescence of the extraordinarily long WS_2_ nanotubes were investigated. Finally, using the ultralong WS_2_ nanotubes, a paper-like material or felt was fabricated. The usual way for assembling carbon nanotube-based buckypaper is through a wet-laying process,^32^ which was modified for fabricating WS_2_ nanotube-based buckypaper. Such inorganic nanotube-based felts would be highly beneficial in electronics, catalysis, sensors, functional electrodes, composites, phase separations, or liquid and air filtrations.^33−40^

Methods and the characterization tools are described in the Supplementary Experimental part.

Particles of tetragonal hydrogen tungsten bronze H_*x*_WO_3_ (1 g; *x* = 0.23–0.33) were sealed under high vacuum (1 × 10^–5^ Pa) in a quartz ampule (Figure S1a; inner and outer diameters 9 and 12 mm, respectively; 130 mm long). The XRD analysis of the bronze H_*x*_WO_3_ powder is shown in Figure S2a. The deep dark blue powder was evenly spread in the ampule and annealed in the horizontal furnace at 800 °C for 30 min. A compact layer of deep blue material (Figure S1b) was obtained, which was later characterized as ultralong nanowhiskers of W_5_O_14_.

The prepared raw material was placed into a quartz boat, and subsequently sulfidated under the flow of H_2_S and H_2_ at 845 °C for 6 h according to the reaction protocol published previously.^31^

Two different processes were tested for the preparation of the buckypaper: the wet-laying of pristine ultralong WS_2_ nanotubes and the wet-laying of ultralong tungsten suboxide nanowhiskers, followed by high-temperature sulfidation treatment. In a typical preparation procedure, 1 g of the selected material (nanowhiskers or nanotubes) was dispersed in 1 L of water using an ultrasonic treatment in a standard laboratory ultrasonic bath. Blue and gray suspensions were gradually filtered through a Durapore HVLP 0.45 μm hydrophilic membrane, respectively. A common laboratory vacuum-assisted filtration setup was used. After the filtration was completed an additional 10 mL of isopropyl alcohol was added to facilitate drying the specimen in air. In the case of deposited tungsten suboxide nanowhiskers the layer was carefully peeled off the filtration membrane and subsequently sulfidated as described below. In such a way, a free-standing WS_2_ nanotube-based paper-like material was obtained. In a similar way, regular WS_2_ nanotubes (average length less than 5 μm and aspect ratio of 100) were deposited on the filtration membrane for comparison, which however did not yield a self-supporting “buckypaper” (*vide infra*).

Gold nanoparticles dispersed in water were prepared by reduction of HAuCl_4_ (30 mg dissolved in 200 mL of water) by borohydride (50 mg dissolved in 50 mL water). Both solutions were mixed by simple pouring. The pink-violet solution containing gold nanoparticles (4.9 ± 1.5 nm in diameter) prepared in such a way was dripped onto the buckypaper. The pristine gold solution and the filtrated liquid were analyzed via absorption measurements.

The growth of the precursor WO_3–*x*_ nanowhiskers was studied extensively in the past.^41−45^ Favorably, the reaction products appear on the surface of oxidized metal tungsten or tungsten oxide powder itself. The 1D growth is based on the volatilization of the well documented WO_2_(OH)_2_ clusters^46−48^ and their redeposition on the tip of the nascent nanowhisker, usually W_18_O_49_. For such transformation, a horizontal flow reactor or vacuum chamber is used in which high temperature annealing under vacuum or hydrogen gas flow is used, respectively. In both systems, the reactive volatile species (WO_2_(OH)_2_) is swept away from the desired substrate location and redeposited on the tip of the W_18_O_49_ nanowhisker. Therefore, such a reaction pathway leads to relatively short W_18_O_49_ nanowhiskers (generally up to ten micrometers) as described in a previous work.^3^ This ascertainment led to the hypothesis of a closed-system reactive growth. For effective preparation of very long nanowhiskers, it is key to have a simultaneous source of tungsten oxide and hydrogen for promoting the oxide volatility. The closed system permits a continuous reaction without any outlet of the reactant species. Here, water molecules, which are both surface-adsorbed and self-produced in the reduction reaction, serve as a shuttle to transport the WO_3–*x*_ molecules from one particle and redeposit it on the tip of the nanowhisker. Favorably, the hydrogen tungsten bronze^49^ (H_0.23–0.33_WO_3_) was chosen as the precursor for the high-temperature reaction in the closed quartz ampule. The blue material obtained in the reaction was investigated by SEM, TEM, and powder XRD. SEM analysis showed a film of agglomerated particles covered and interconnected by a dense web of ultralong curved nanowhiskers (Figure 1a, lower magnified overview in Figure S3, length >100 μm, average diameter 53 ± 27 nm). Interestingly, the extraordinarily extended nanowhiskers were present mostly on the surface of the agglomerates. Deeper in the porous layer, the whiskers were substantially shorter. XRD analysis (Figure S2b) showed the presence of the W_5_O_14_ (W_20_O_56_) phase (ICDD PDF 00-041-0745) and W_20_O_58_ (ICDD PDF 04-007-0501), discussed in detail in the SI. Due to the extreme aspect ratio of the nanowhiskers (>1000), some diffraction peaks are bland or completely suppressed by the relative intensities of the (001), (600), and (540) signals.^50^ In contrast with the results from the horizontal flow reactor, where W_18_O_49_ nanowhiskers were predominant, the reduction here was presumably milder and limited by the hydrogen content or the overpressure in the ampule. The internal pressure was approximated at 430 kPa, determined by the hydrogen content and the associated water content derived from the tungsten hydrogen bronze. TEM analysis (Figure 1b) showed a typical tungsten suboxide nanowhisker pattern consisting of lines along the [001] axis and perpendicular layers consisting of [WO_6_] octahedra. Further, selected area electron diffraction (SAED) analysis was performed, displaying a pattern of monoclinic WO_3–*x*_ nanowhiskers (Figure 1c). The distance between the layers of the polyhedra in the direction ⟨001⟩ was 0.38 nm. Moreover, the cross-like SAED pattern corresponds to indices 001 and 600 (marked by yellow dots), which is in accordance with the literature.^50^ Cross-sectioning and STEM-HAADF analyses were carried out in order to directly confirm the ultralong nanowhisker structural nature. The analysis confirmed the findings of the XRD measurements of the bulk sample, i.e., the lamella structure fitted the arrangement of the W_5_O_14_ phase in the nanowhiskers (Figure 1d).^50^

**Figure 1:**
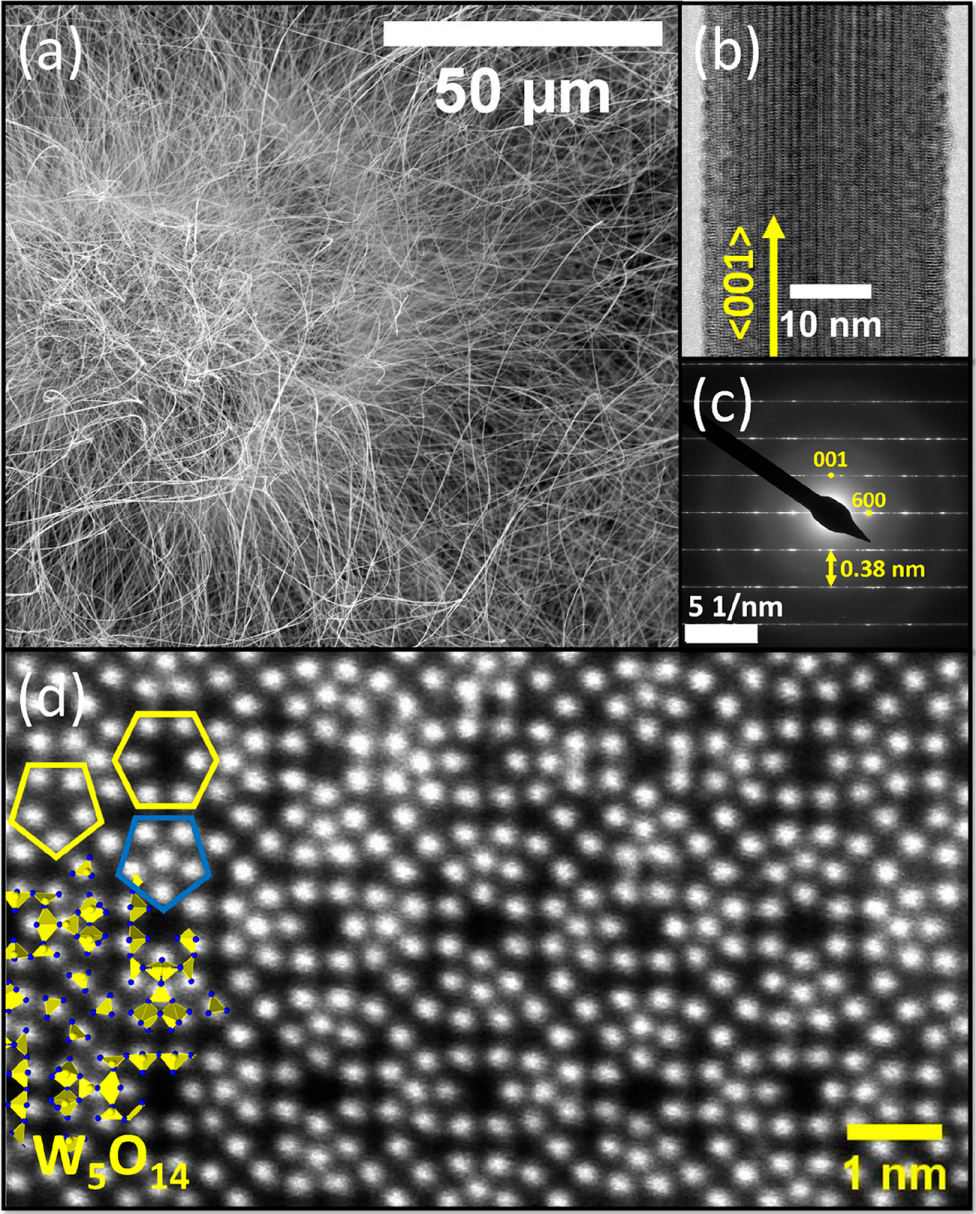
Electron microscopy analysis of WO_3–*x*_ nanowhiskers grown from hydrogen tungsten bronze particles. (a) SEM image of the ultralong nanowhiskers grown on the surface of the agglomerated oxide bulk. (b) Detailed HRTEM measurement of the nanowhisker (diameter of approximately 30 nm). The yellow arrow indexes the [001] axis. (c) Selected area electron diffraction of the W_5_O_14_ nanowhisker in (b). The distance (0.38 nm) between layers of the polyhedra corresponds closely to the tungsten suboxide nanowhisker arrangement. Point diffractions forming notional cross fit to (001) and (600). (d) Cross-section of the ultralong nanowhisker reveals a W_5_O_14_ lattice (indicated on the left side) from the ⟨001⟩ direction. The structure consists of multiple hexagonal and pentagonal channels (yellow polygons) formed by [WO_6_] octahedra.^50^ Another structural feature is pentagonal columns (blue pentagon) formed by the line of pentagonal bipyramids [WO_7_].

The film of nanowhiskers was carefully transported into a horizontal reactor and sulfidated under the flow of a H_2_S and H_2_ mixture. The initially blue layer changed color to greenish brown, typical for WS_2_. The SEM analysis (Figure 2a and overview in Figure S4) revealed intact morphology of the layer consisting of ultralong WS_2_ nanotubes and also nanobelts (length >100 μm), which were further inspected by TEM. The preparation of the specimen for TEM examination was accomplished by cautious wiping of the material surface by the TEM grid. Subsequent analysis showed indeed extraordinarily long WS_2_ nanotubes (Figures 2b, S5, S6), which preserved the morphology and curvature of its precursor W_5_O_14_ nanowhiskers. The SAED measurement (Figure 2c) of the nanotube shown in Figure 2b indicated a typical pattern for layers in the armchair conformation (30°) and chiral layers with 15° tilt. Some of the nanotubes were 300 μm and perhaps even longer. As vindicated by the TEM measurements, frequently the ends of the nanotubes were tattered due to the detaching of the specimen from the nanotube web on the original material film (Figure S5), i.e., the original nanotubes were longer than seen. In some cases, the nanotube’s bending was compensated by the formation of wrinkles (Figure S6) on the inward side of the nanotube wall. Deformations of this kind were observed in the past during *in situ* micromanipulation in TEM and were attributed to compression strain, which led to a buckling transition of the layers.^51,52^ Moreover, other WS_2_ morphologies were present, mainly nanowires (in other words, nanotubes with a filled core) or nanobelts, which could be associated with collapsed nanotubes, all exceptionally long (Figure S7). Nanoribbons obtained from collapsed nanotubes were studied in the past using Raman spectroscopy.^53^ Based on TEM analysis, the content of nanotubes in the sample could be estimated as 70–80%.

**Figure 2:**
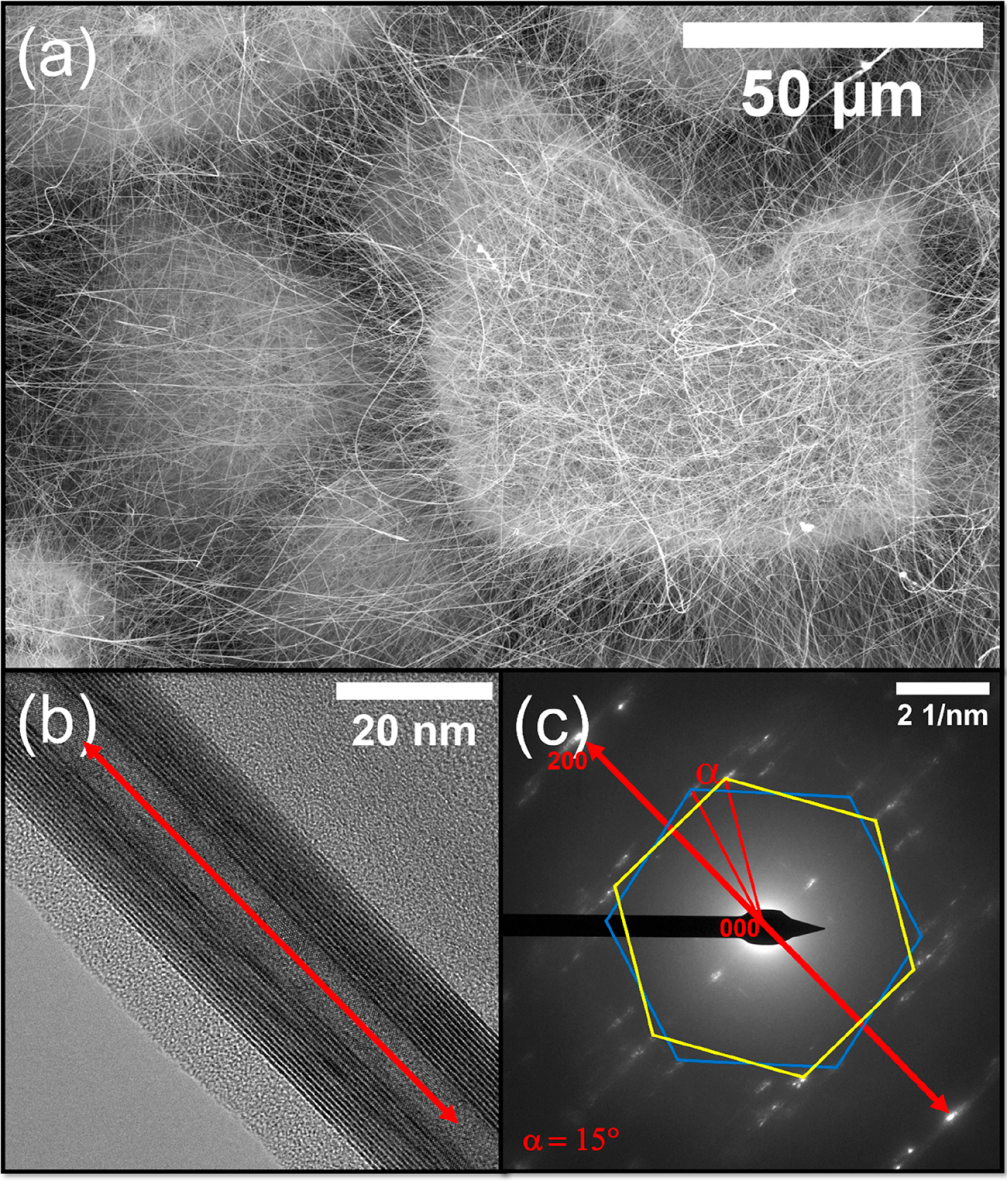
Electron microscopy analysis of WS_2_ nanotubes produced by sulfidation of the W_5_O_14_ nanowhiskers. (a) SEM image of ultralong WS_2_ nanotubes. (b) TEM image of the WS_2_ nanotube with a marked axis along the cavity direction. The interlayer distance was measured as 0.625 nm. (c) Corresponding SAED measurement typical for the hexagonal structure of the WS_2_ nanotube in chiral (blue hexagon, approximately 15°) and armchair conformation (yellow hexagon) of the WS_2_ layers.

Four-probe electrical measurements (Figure 3a) performed on an ensemble of 8 nanotubes yielded conductivities within the range from 1.5 S m^–1^ to 250 S m^–1^. Assuming mobility of 50 cm^2^ V^–1^ s^–1^ (previously estimated in different WS_2_ nanotubes^54,55^), the carrier concentrations within these long WS_2_ nanotubes span the range between (10^15^–10^17^) cm^–3^. There was no difference in *IV* curves measured in the dark and ordinary sunlight (in 4-contact measurements). These characteristics are comparable to the previously reported ones for much shorter WS_2_ nanotubes^54,55^ and slightly outperform multilayer WS_2_ devices.^56^

**Figure 3:**
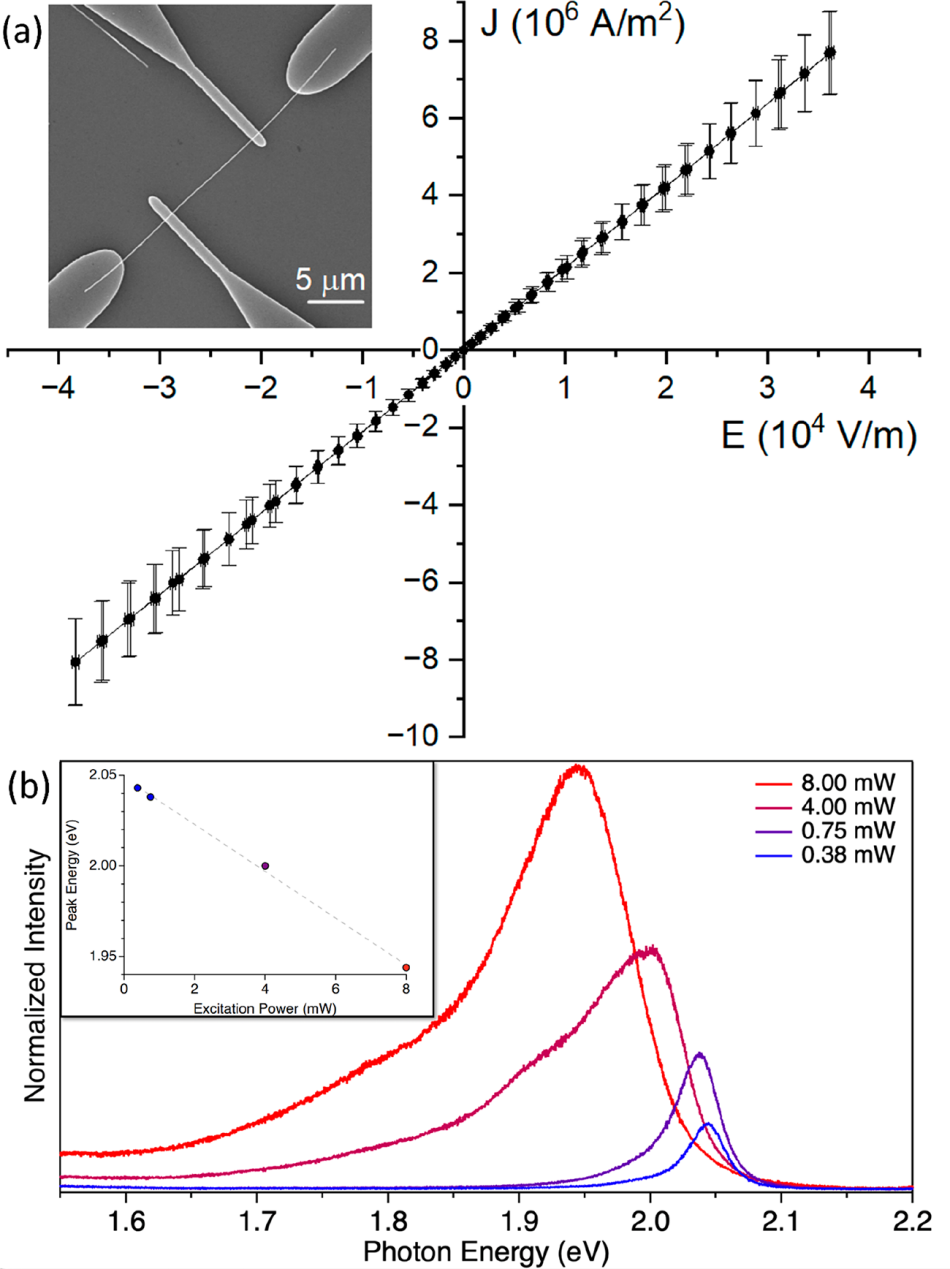
(a) Electrical characterization of a single nanotube. The acquired Ohmic *IV* characteristic is shown, as obtained on a WS_2_ nanotube with a mean diameter of (72 ± 5) nm and the length of the channel between the inner contacts (9.93 ± 0.09) μm, yielding the conductivity of (2.10 ± 0.29) × 10^2^ S m^–1^. The inset shows the typical geometry of a fabricated device used for the measurement. (b) Photoluminescence spectra of the WS_2_ ultralong nanotubes as a function of the laser intensity.

The photoluminescence (PL) spectra of the nanotubes at different excitation intensities were collected (Figure 3b). The PL peak ascribed to the direct gap exhibits a redshift of about 100 meV when the light intensity is increased 20 times (see inset of Figure 3b). Assuming ∼5 × 10^–4^ eV/deg change in the bandgap energy vs the temperature,^57^under the strongest beam intensity the sample was heated to about 200 °C above room temperature due to the laser beam irradiation. At the highest excitation intensities (50 and 100%), the line shape of the PL becomes less symmetric below the bandgap emission and a shoulder is observed at some 100 meV below the main peak. This shoulder can be possibly attributed to the radiative recombination of trions.^58^

The ultralong WS_2_ nanotubes were investigated as building blocks for assembling paper-like materials or felts. Two different approaches were tested. Initially, the as-prepared WS_2_ nanotubes were dispersed in water and filtered through a hydrophilic PVDF membrane. After drying in air, a gray layer was obtained, photographed, and analyzed by SEM (Figure 4a). The film was found to be difficult to peel-off from the PVDF membrane in one piece. Cutting carefully with a scalpel a piece of this “buckypaper” the stand-alone film curled (see Figure 4a). The buckypaper film attached to the membrane filter (Figure 4a inset) was found to be mechanically stable and could be bent without any breaking or release of a powder. However, to get a freestanding WS_2_ nanotube paper-like material, a different approach was developed. Tungsten suboxide nanowhiskers were similarly dispersed in water and filtered through the PVDF membrane. After drying out in air a dark blue film was obtained, which was separated from the membrane (1 × 2 cm^2^). Subsequently, it was sulfidated in the tube furnace by the same procedure used for pristine nanowhiskers. The resulting dark gray piece was crumpled and somewhat twisted by the heat treatment yet was free-standing (Figure 4b and inset). Alternatively, a free-standing flat film was produced by sulfidation of the tungsten oxide material sandwiched between two quartz disks. Detailed SEM analysis of both materials showed very similar morphologies (Figure 4c,d). Attempts to produce films of this kind from much shorter tubes (usually less than 10 μm) described previously^2^ were unsuccessful. Such deposited layers exhibited poor compactness and mechanical stability; see Figure S8. Therefore, the ultralong WS_2_ nanotubes described in this work were found to be vital for the formation of a mechanically stable “buckypaper”.

**Figure 4:**
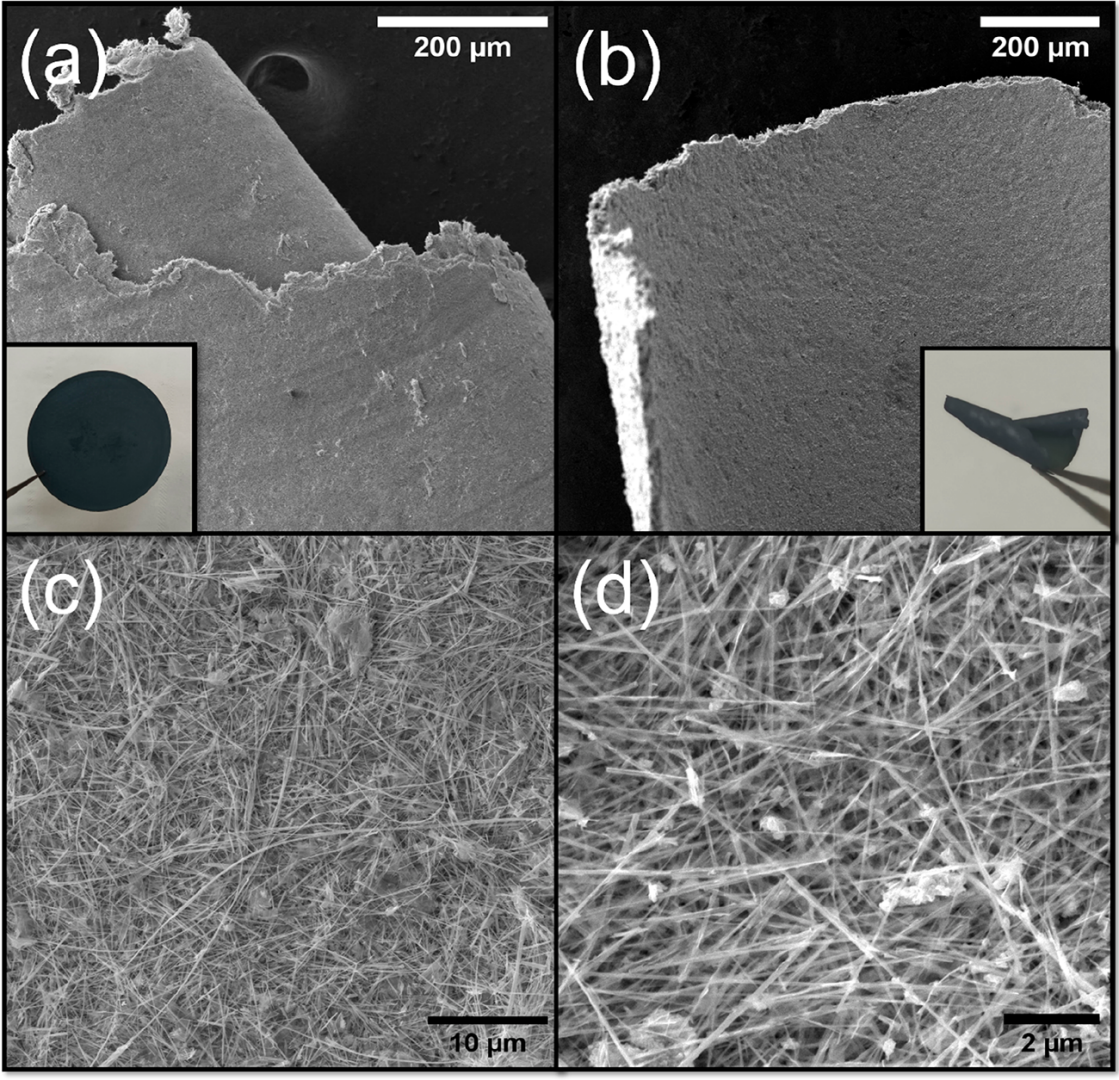
SEM analysis of wet-laid-prepared materials. The water suspension of pristine WS_2_ ultralong nanotubes was filtered through the filtration membrane and formed a uniform layer of WS_2_ inorganic nanotubes (a). The image of the membrane with assembled WS_2_ paper-like material is displayed in the inset. In (b) the sulfidated wet-laid layer of W_5_O_14_ nanowhiskers is displayed. During the high-temperature treatment the layer was twisted as displayed in the inset. Detailed SEM images show the morphology of the prepared layers by assembly of pristine nanotubes and sulfidated layer of nanowhiskers in (c) and (d), respectively. Thanks to the extraordinarily long nanotubes, interconnected and mechanically stable felts or paper-like materials could be established.

Finally, the WS_2_ “buckypaper” deposited on the PVDF membrane (Figure 4a inset) was tested as a filter for removing gold nanoparticles dispersed in water (see Figure 5a). Tungsten disulfide is a well-known material for its affinity to metallic nanoparticles.^59−61^ Therefore, the WS_2_ buckypaper could act as a highly efficient filter and adsorbent. Indeed, the color change of the filtered solution from pink-violet to colorless indicated successful nanoparticle removal by the filter. Further, the solution and filtrate were subjected to analysis by UV–vis spectroscopy (Figure 5b). Based on the diminution of the absorption signal at 528 nm in the filtrate, the yield of (one round) filtration could be estimated at approximately 97%, which could be further improved by more rounds of filtrations, and by further optimization of the process for the preparation of the buckypaper.

**Figure 5:**
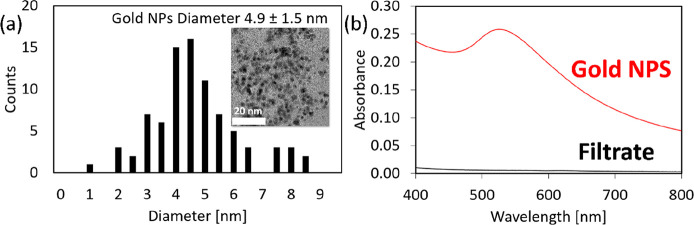
Gold nanoparticles filtration on the WS_2_ buckypaper membrane. The gold nanoparticles were estimated to be approximately 4.9 ± 1.5 nm in diameter by measurement of 50 individual nanoparticles on a TEM grid (a). The colloidal water solution of the gold nanoparticles was filtered through the WS_2_ nanotube buckypaper. (b) Filtration efficiency was estimated to be 97% based on the change in absorbance (528 nm). The mechanism of the filtration could be explained based on the affinity of metal nanoparticles to the outer sulfur atoms in WS_2_ nanotubes.

In conclusion, selective growth of ultralong W_5_O_14_ nanowhiskers was accomplished through annealing of H_0.23–0.33_WO_3_ bronze in a sealed ampule. The ultralong nanowhiskers were sulfidated in a reducing atmosphere and converted into mostly WS_2_ nanotubes. Visibly, the nanotubes on the surface of the agglomerates were 100 to 300 μm long or even longer. The reaction was well-controlled, and hence the long nanotubes were obtained reproducibly in high yields. The present work paves the way for the systematic use of such long nanotubes for advanced applications, in particular, polymer nanocomposites for medical technologies and optoelectronic devices. Wet-laying processes were used to prepare WS_2_ nanotube-based paper-like materials. This buckypaper can be used for a variety of applications, like ultrafiltration^61a^ of waste and colloids, and in the future possibly for renewable energy applications and remediation of environmental hazards.

## Supporting Information Available

Powder X-ray diffraction, electron microscopy, electrical characterization, and photoluminescence measurements (PDF)
