# Supplementary material for: Submillimeter-Long WS2 Nanotubes: The Pathway to Inorganic Buckypaper
Source: Nano Lett. 2023 Oct 8;23(22):10259–66. doi: 10.1021/acs.nanolett.3c02783 (PMC10683059; doi:10.1021/acs.nanolett.3c02783)
Supplement: Supplementary file 1 — nl3c02783_si_001.pdf [file nl3c02783_si_001.pdf]

Supplementary information for:

Submillimeter-long WS_2 nanotubes: the pathway to inorganic buckypaper

Vojtěch Kundrát[a,b], *Rita Rosentsveig*[a], *Kristýna Bukvišová*[b,c], *Daniel Citterberg*[c], *Miroslav Kolíbal*[c,d], *Shachar Keren*,[e] *Iddo Pinkas*,[f] *Omer Yaffe*,[e] *Alla Zak*,[g] *Reshef Tenne*[*a]

[a] Department of Molecular Chemistry and Materials Science, Weizmann Institute of Science, Rehovot 7610001, Israel

[b] Thermo Fisher Scientific, Vlastimila Pecha 12, CZ-62700 Brno, Czech Republic

[c] Central European Institute of Technology, Brno University of Technology, Purkynova 123, CZ-61200 Brno, Czech Republic

[d] Institute of Physical Engineering, Brno University of Technology, Technická 2, 616 69 Brno, Czech Republic

[e] Department of Chemical and Biological Physics, Weizmann Institute of Science, Rehovot 7600001, Israel

[f] Department of Chemical Research Support, Weizmann Institute of Science, Rehovot 7600001, Israel

[g] Faculty of Science, Holon Institute of Technology, Golomb Street 52, Holon 5810201, Israel

*Corresponding author. E-mail: reshef.tenne@weizmann.ac.il

Supplementary Experimental part

Powder X-ray diffraction

X-ray powder diffraction (XRD) was performed using TTRAX III (Rigaku, Tokyo, Japan) theta-theta diffractometer. The set-up was equipped with a rotating copper anode X-ray tube operating at 50 kV/200 mA. The samples were prepared on a zero-background Si substrate. They were scanned using the Cu Kα line (wavelength 1.54 Å) in specular diffraction mode (θ/2θ scans) from 10-80° (2θ) with a step size of 0.02° and a scan rate of 0.5° per min.

Electron microscopy

Scanning electron microscopy (SEM) analyses were performed using Thermo Fisher Scientific Apreo 2S SEM.

Cross-sections of the nanotubes for transmission electron microscopy (TEM) were prepared using a Thermo Fisher Scientific Helios - NanoLab 660. The intended region of interest was covered with a layer of amorphous carbon using electron beam-assisted deposition. An additional protecting layer was subsequently added using ion beam-assisted deposition. A chunk of deposited material containing the nanotube cross-section was transferred to a TEM copper grid according to a standard lift-out procedure.

TEM analysis was performed using a Thermo Fisher Scientific Talos F200i operated in high resolution (HRTEM) mode at a high accelerating voltage of 200 kV and a beam current of 1 nA. HRSTEM cross-sectional analysis was done using Thermo Fisher Scientific Talos F200X equipped with X-CFEG at 200 kV and beam current 50 pA. TEM images were post-processed using the Velox and ImageJ software.

Electrical Characterization

The ultralong WS_2 nanotubes were meticulously transferred onto a boron-doped Si wafer, which was covered with a SiO_2 layer approximately 115 nm thick. Selected nanotubes were contacted by electron beam lithography, utilizing an AR-P 679.04 (PMMA) resist (~800 nm thick). For the electrical contacts, Cu (~350 nm) and Au (~20 nm) layers were evaporated using Ti (~10 nm) as an adhesion layer (total thickness ~ 380 nm) onto the developed sample, followed by a lift-off process. The electrical characterization was done at room temperature and atmospheric pressure utilizing a Cascade Microtech MPS 150 probe station and Keithley 4200-SCS parameter analyzer. The diameter of the tubes varies slightly along their growth axis (by approx. 25 % maximum, as inferred from SEM images). Hence, the mean diameter value was used for the calculation of current density and conductivity. TEM analysis revealed that the nanotube's interior constituted approximately 70 % of WS_2 layers and 30 % of hollow cavity space. The calculations were performed accordingly to this ratio of hollow space and WS_2 walls. Control leakage current measurements on the contacted nanowires have shown four orders of magnitude lower values within the investigated voltage range.

Photoluminescence measurement

PL measurements of the long WS_2 nanotubes were carried out on a Horiba LabRAM HR evolution micro-Raman system built around an Olympus BXFM modular microscope. The measurements were done using a 532 nm laser through 100x MPlanFL N NA = 0.9 (RT - 100% = 2.5mW, 50% = 1.25mW, 10% = 0.225mW), 50x LMPlanFL N NA = 0.5 (LWD) (LT - 10% = 0.4mW), and 10X MPlan N NA = 0.25 (RT - 100% = 8mW, 50% = 4mW, 10% = 0.75mW) objectives. The PL spectra were dispersed on a 600gr/mm grating in an 800mm spectrograph with high resolution and low stray light. The low-temperature measurements utilized a Linkam LN2 THMS temperature-controlled stage.

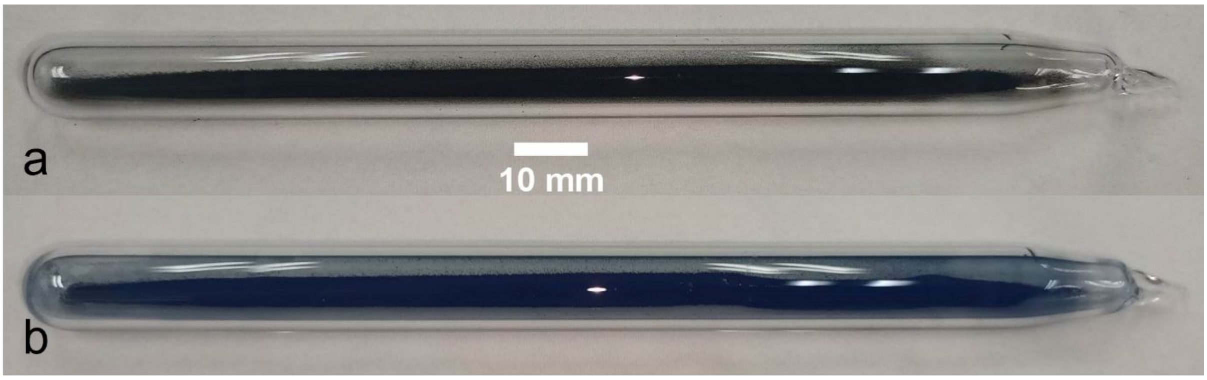

Figure S1. Photography of ampule before **a)** and after **b)** reaction. **a)** the dark blue/black powder of $H_{0.23-0.33}WO_3$ was evenly spread in the ampule. **b)** after the reaction the material was bright blue and in a compact layer slightly fixed to the ampule.

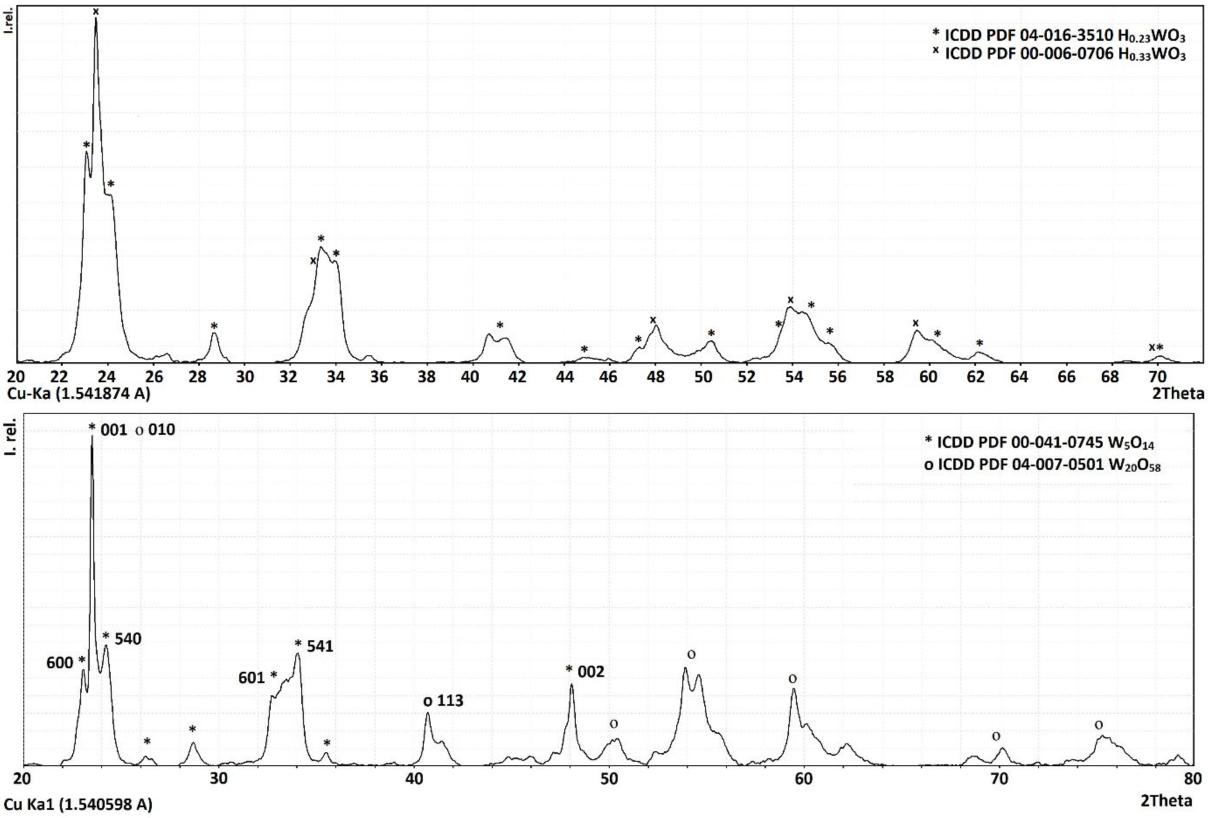

Figure S2. Powder X-ray analyses of the powder precursor **a)** and resulting nanowhisker layer **b)**. **a)** The hydrogen tungsten bronze used as a precursor consisted of two tetragonal phases with different stoichiometries $H_{0.23}WO_3$ (ICDD PDF 04-016-3510) and $H_{0.33}WO_3$ (ICDD PDF 00-006-0706). **b)** The high-temperature transformation resulted in the monoclinic phases W_5O_{14} (ICDD PDF 00-041-0745) and $W_{20}O_{58}$ (ICDD PDF 04-007-0501). The diffractograms overlap to some degree with the major diffractions being identical to both structures. Therefore, XRD analysis alone was not able to give a definitive answer on the nature of the crystalline phase of the nanowhisker. HRSTEM analysis of the nanowhisker cross-section provided a definite confirmation of the W_5O_{14} phase. Possibly, the particles forming the bulk of the agglomerate (see **Figure S3**) belong to the $W_{20}O_{58}$ phase, whereas the ultralong nanowhiskers on the surface are W_5O_{14}.

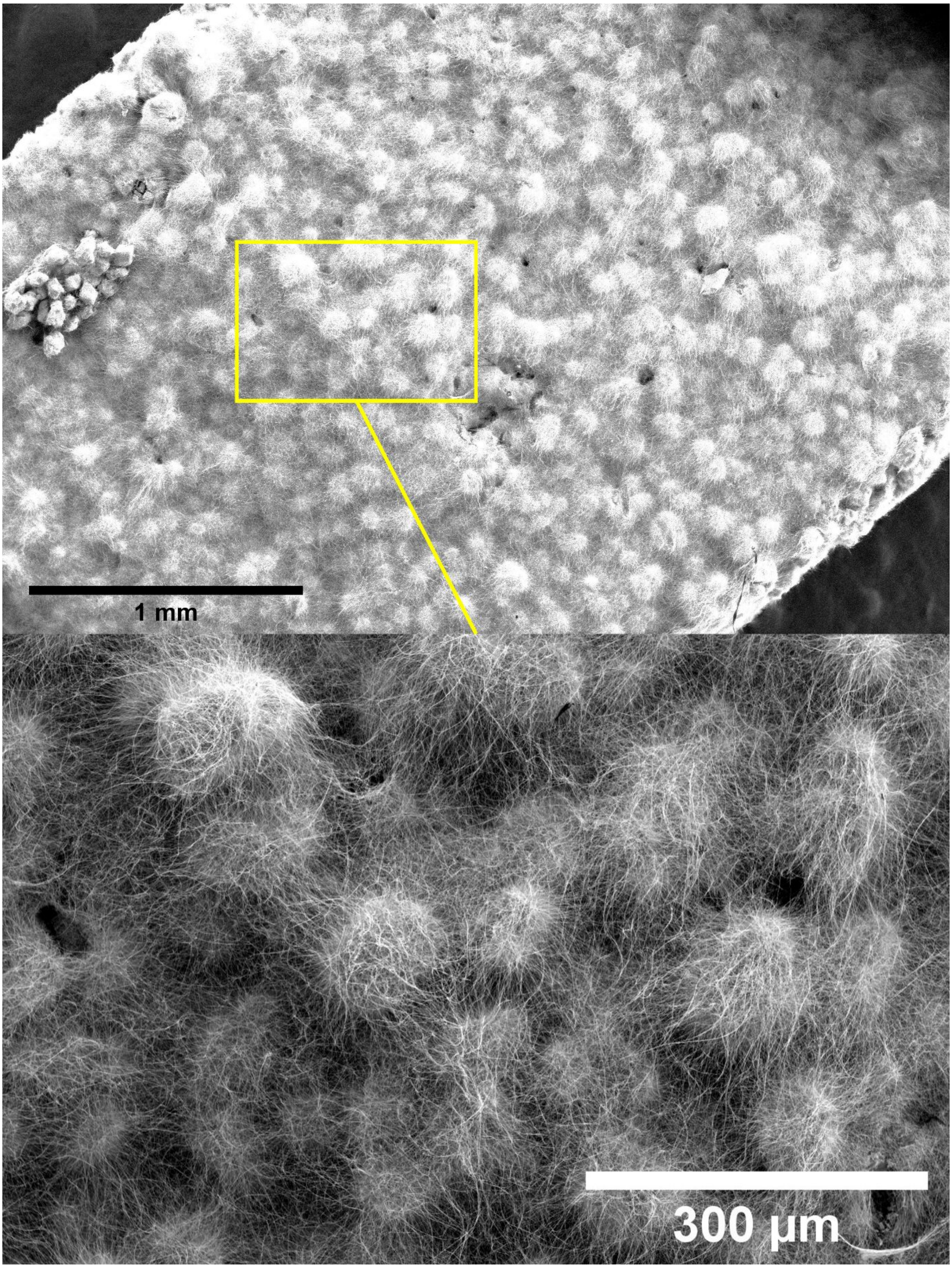

Figure S3. Low magnification SEM image of the agglomerates with the W_5O_{14} nanowhiskers on the surface.

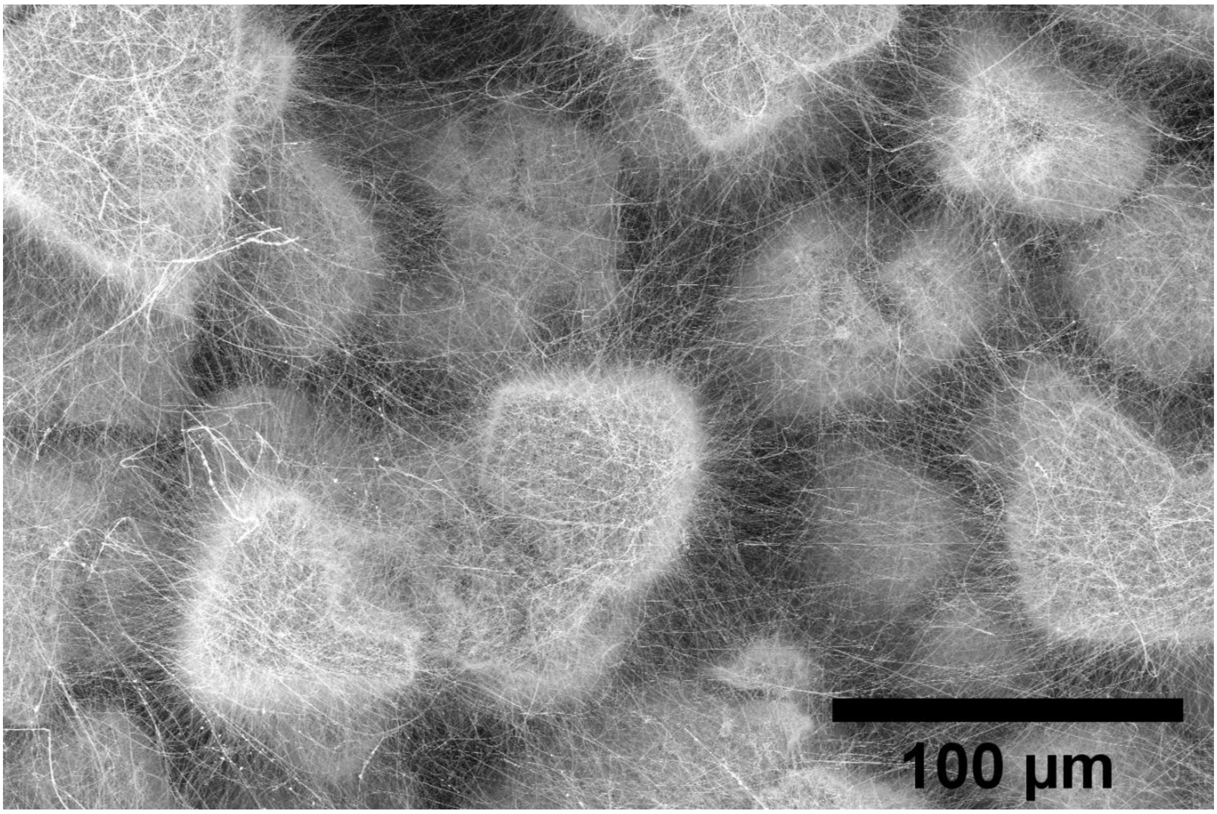

Figure S4. Low-magnification SEM image of the WS_2 nanotube film. Clearly, some of the nanotubes reach a fraction of a millimeter.

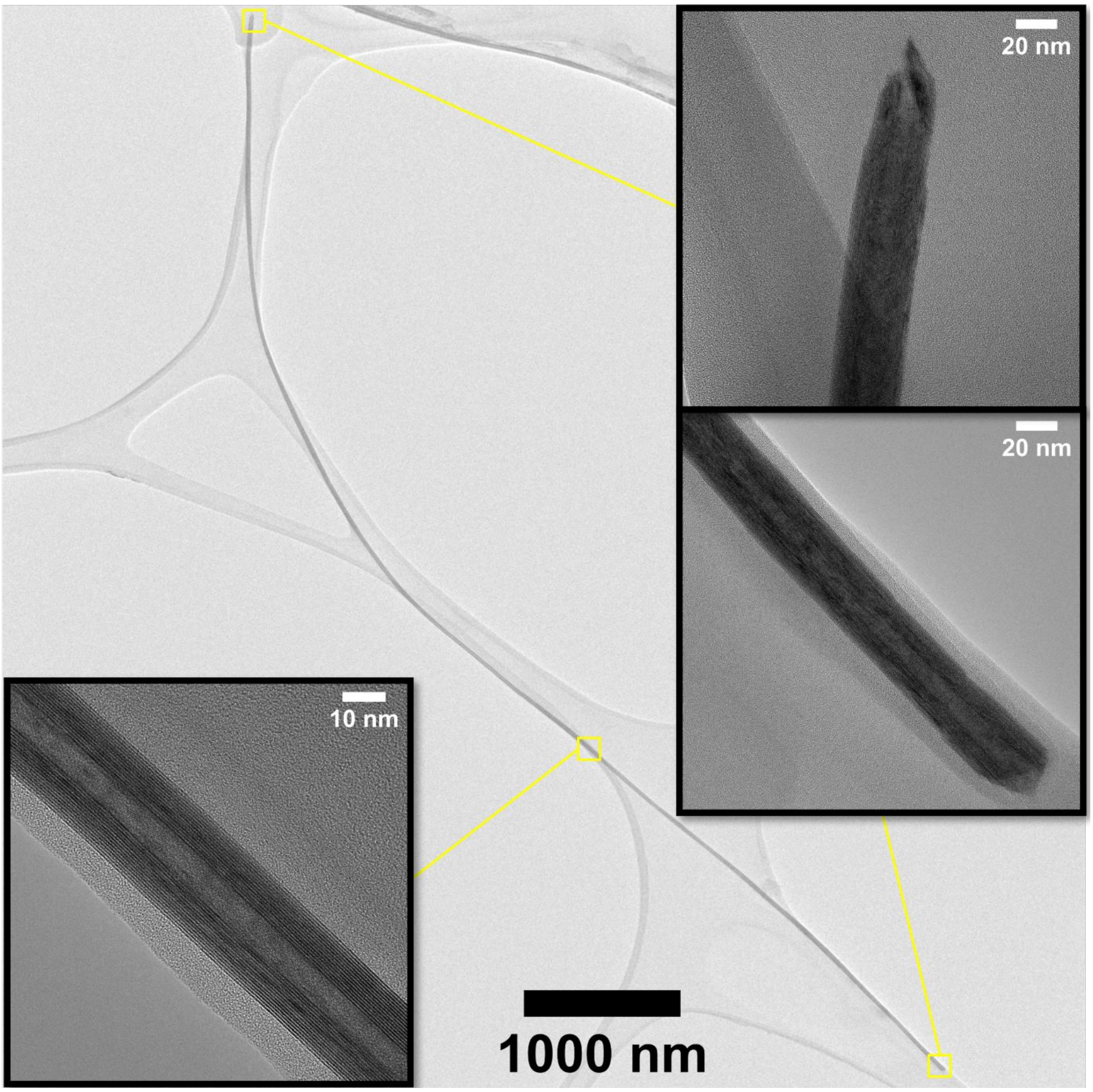

Figure S5. TEM analysis of the ultralong WS_2 nanotube fragment ripped out from the web during the grid preparation. The nanotube was inspected along its profile showing its morphological consistency. The tips of the fragment are jagged due to the tearing from the web.

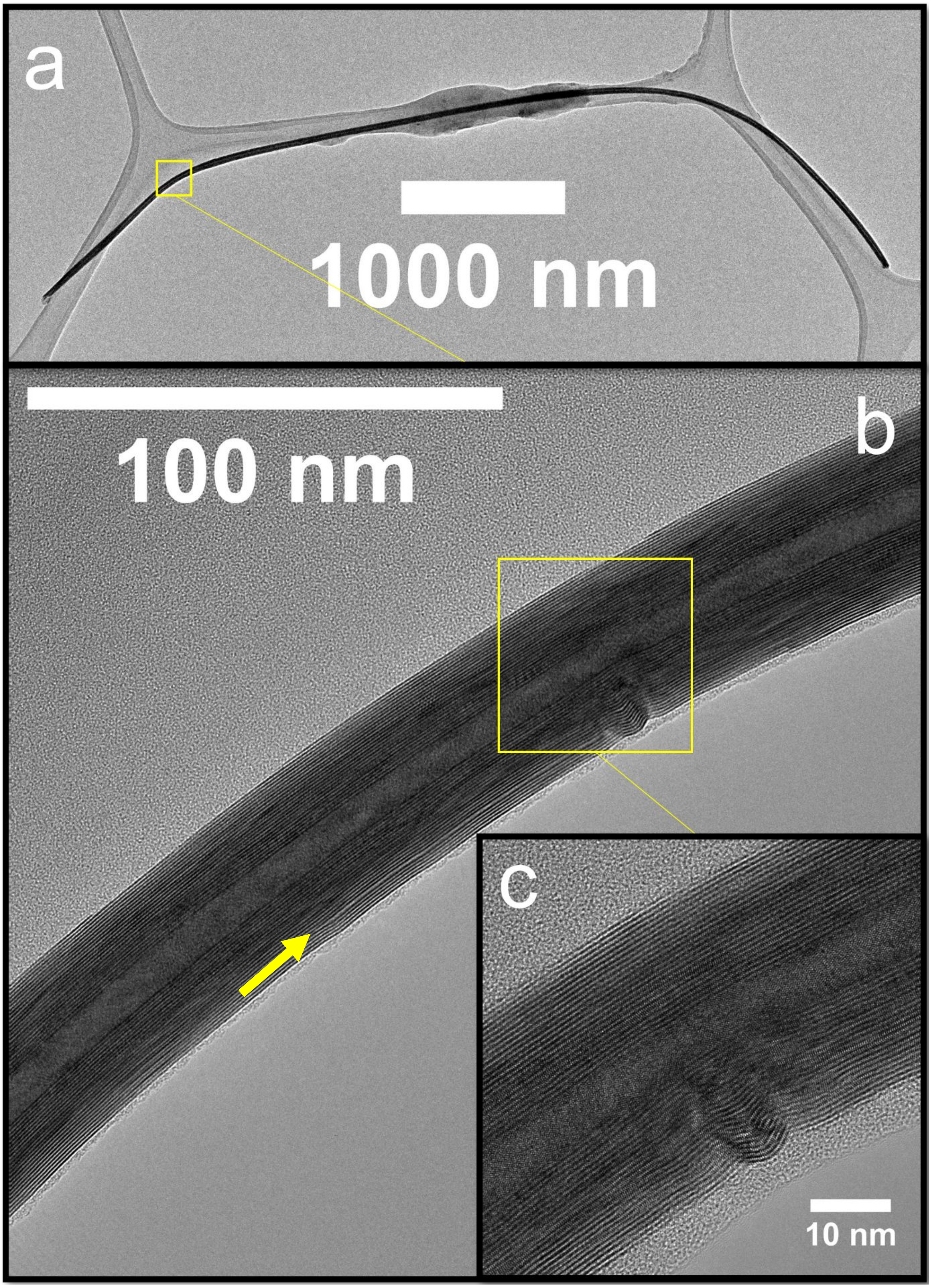

Figure S6. a) TEM image of the curved nanotube. **b)** magnified portion of the curved nanotube from **a)** showing wrinkles in the concave layers buckled under a compression strain. The yellow arrow indicates the slight peeling off of the outer layers and minuscule wall cavity formation. **c)** magnified WS_2 layer wrinkle

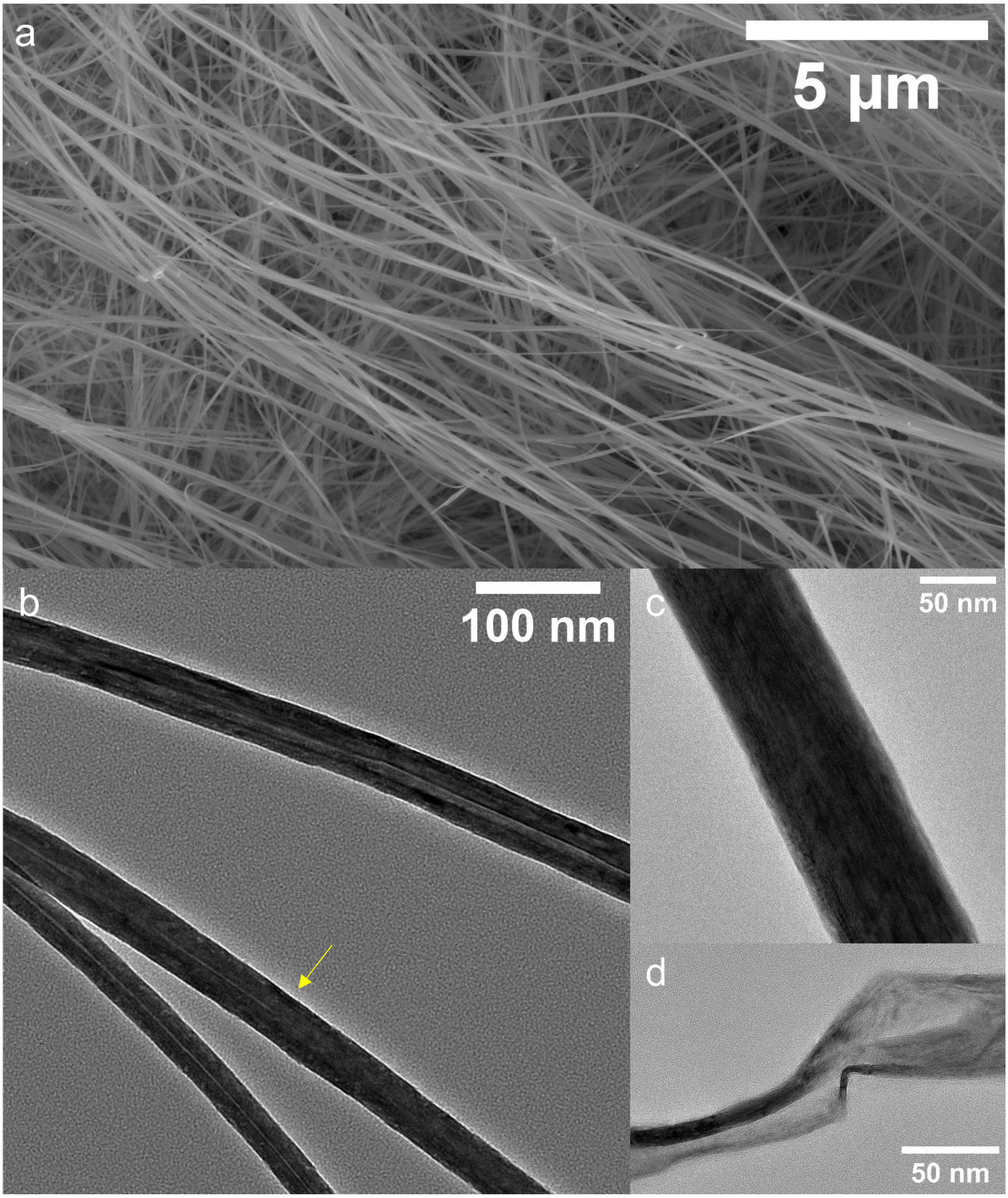

Figure S7. Electron microscopy measurements of various other WS_2 morphologies in the sulfidated layer. **a)** SEM image of a mixture of WS_2 nanotubes, belts, and whiskers, usually found between the particles in the layer. In a number of cases the morphology of the specimen changes along its length, e.g. from a nanotube to a nanobelt and back, primarily induced by bending and strain. A similar example can be observed in **b)** marked by the yellow arrow. The nanotube cavity collapses into a WS_2 nanowhisker and later reappears again. **c)** example of WS_2 nanotube filled in the core by additional WS_2 layers. **d)** segment of the WS_2 nanobelt formed by bending and collapsing of the WS_2 nanotube.

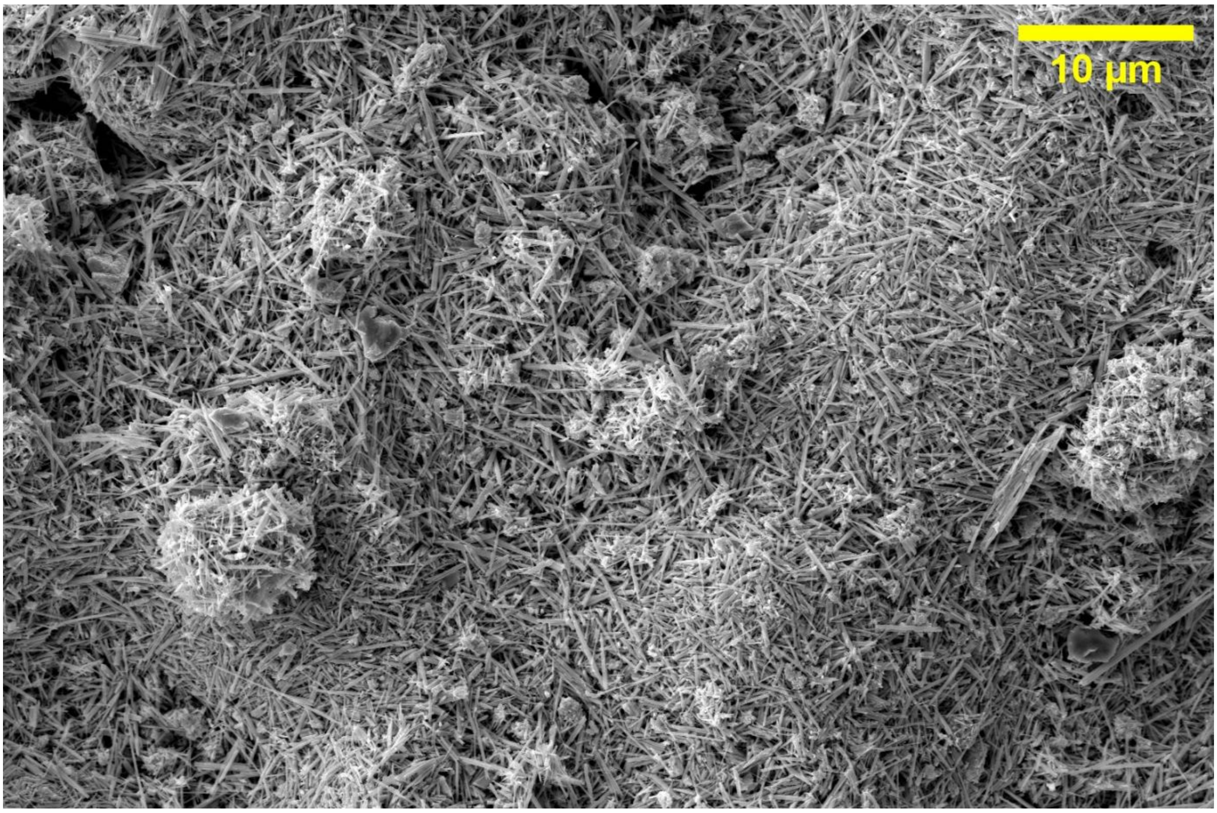

Figure S8. SEM image of wet-laid regular WS_2 nanotubes (app. 5 μm long) showing a filtration cake, which was unable to arrange itself into a self-sustained paper or felt. When scratched, the layer disintegrated to powder, leaving a distinct black stain on the office paper. This showed the poor integrity of the film in comparison with the self-supporting paper prepared with ultralong WS_2 nanotubes (see **Figure 4** in the main text).
